# Inflammatory Bowel Diseases: When Natural Friends Turn into Enemies—The Importance of CpG Motifs of Bacterial DNA in Intestinal Homeostasis and Chronic Intestinal Inflammation

**DOI:** 10.4061/2010/641910

**Published:** 2010-08-16

**Authors:** Florian Obermeier, Claudia Hofmann, Werner Falk

**Affiliations:** Department of Internal Medicine I, University of Regensburg, 93042 Regensburg, Germany

## Abstract

From numerous studies during the last years it became evident that bacteria and bacterial constituents play a decisive role both in the maintenance of intestinal immune homeostasis as well as in the development and perpetuation of chronic intestinal inflammation. In this review we focus on the role of bacterial DNA which is a potent immunomodulatory component of the bacterial flora. Bacterial DNA has been shown to be protective against experimental colitis. In contrast bacterial DNA essentially contributes to the perpetuation of an already established chronic intestinal inflammation in a Toll-like receptor (TLR)9-dependent manner. This dichotomic action may be explained by a different activation status of essential regulators of TLR signaling like Glycogen synthase kinase 3-*β* (GSK3-*β*) depending on the pre-activation status of the intestinal immune system. In this review we suggest that regulators of TLR signaling may be interesting therapeutic targets in IBD aiming at the restoration of intestinal immune homeostasis.

## 1. Introduction

Chronic intestinal inflammation as found in Inflammatory Bowel Diseases (IBD) is common in developed countries with a prevalence of about 0.5% and, according to the chronicity and the early onset of disease, has an enormous socioeconomic impact. Despite great advances in the understanding of IBD in the past ten years the aetiology of both Ulcerative Colitis (UC) and Crohn's disease (CD) remains elusive.

The spectrum of possible explanations reaches from the autoimmune hypothesis which seems to be supported by the existence of different autoantibodies against epithelial cells in IBD (reviewed in [1]) to the statement that IBD should be considered as an infectious disease [2] which may develop based on a defective immune response [3, 4].

Although some truth may lay in every specific hypothesis the most likely pathophysiologic explanation according to the current knowledge is that commensal luminal bacteria with which we normally live in friendly or even symbiotic coexistence are mistakenly recognized as possible pathogens resulting in a chronic inflammatory response.

Obviously, both complex environmental [5] and genetic factors [6] contribute to the development of IBD. The most important susceptibility gene described for CD, the NOD2 gene [7, 8], participates in the innate immune response to the bacterial wall component muramyl dipeptide (MDP). Recently, strong genetic interactions between polymorphisms in CD-associated variants of NOD2, IL23R, DLG5, and Toll-like receptor 9 (TLR9, coding for the intracellular receptor which recognizes bacterial DNA) have been demonstrated [9]. Taken together, these data argue for a central role of interactions between bacteria and the mucosal immune system in the pathogenesis of CD. Supporting this view, studies from several murine IBD models underlined the importance of the bacterial flora in the pathogenesis of chronic intestinal inflammation [10].

## 2. Proinflammatory Potential of Bacterial DNA: CpG-Motif-Containing DNA Contributes to the Perpetuation of Chronic Colitis

During the last years, several groups aimed to characterize the role of numerous bacterial constituents in murine colitis models. These studies were put on a new basis by the identification of pattern recognition receptors for microbial constituents including the Toll-like receptors (TLRs). We and others focused on the role of bacterial DNA as a specific bacterial constituent based on its ability to mount a strong Th_1_-skewed immune response resulting in extraordinary high production of tumor necrosis factor (TNF), interferon (IFN)-*γ*, or Interleukin (IL)-6 [11–14], which is also found in experimental and human IBD. Ten years ago, unmethylated cytosine-guanosine (CpG) sequences were identified as the immunostimulatory component of microbial DNA [15], and five years later, TLR9 was identified as the receptor responsible for CpG recognition [16].

In a first approach, we could demonstrate in different models of murine colitis that treatment with CpG-containing oligodeoxynucleotides (CpG-ODN) results in an exacerbation of established intestinal inflammation [17]. Vice versa, TLR9-deficient mice which do not respond to CpG motifs showed a more than 50% reduced chronic intestinal inflammation two months after the induction of chronic dextran sodium sulphate- (DSS-) induced colitis. The latter finding further indicates an important contribution of commensal-derived CpG motifs to the perpetuation of chronic intestinal inflammation. This was further confirmed by a strong therapeutic effect of adenoviral-derived inhibitory motifs in different colitis models, including chronic DSS-induced colitis, the T cell-dependent SCID transfer model of colitis, and IL-10-deficient mice, which develop spontaneous colitis [18]. Together these results support the hypothesis that bacterial constituents and specifically bacterial DNA essentially contribute to the perpetuation of established chronic intestinal inflammation [17].

## 3. Anti-Inflammatory Potential of Bacterial DNA: Protective Qualities of Bacterial DNA in Intestinal Inflammation and Possible Mechanisms

In sharp contrast to the results arguing for a proinflammatory role of bacterial DNA in established intestinal inflammation, we and others found that CpG-ODN exposition had strong protective effects in different models of intestinal inflammation when used in a prophylactic approach. Pretreatment with CpG-ODN resulted in a strong protection from acute DSS colitis as well as from acute Oxazolone- and TNBS-induced colitis [19–21]. At first sight these surprising anti-inflammatory effects of a potent immune stimulating, proinflammatory bacterial compound seem to be confusing.

One important contribution to these protective effects seems to be made by intestinal epithelial cells.

Anti-inflammatory effects of bacterial DNA on intestinal epithelial cells with an inhibition of TNF, and IL-8 secretion as well as NF*κ*B activation were demonstrated in several reports [22, 23]. It was further shown that the anti-inflammatory effects of bacterial DNA/TLR9 interaction on intestinal epithelial cells depend on the origin of the bacterial DNA. In contrast to DNA from pathogenic bacterial strains, DNA isolated from Lactobacillus rhamnosus GG and other probiotic bacteria attenuated TNF-induced NF*κ*B activation and NF*κ*B-mediated IL-8 expression [22–24]. Interestingly, in contrast to the apical stimulation of epithelial cells, basolateral stimulation resulted in a strong NF*κ*B activation and a proinflammatory response [25]. The protective, anti-inflammatory response of epithelial cells upon apical stimulation by DNA derived from commensal or probiotic strains seems reasonable as this is exactly what occurs under physiologic conditions in our intestinal tract. Therefore, apical stimulation of intestinal epithelial cells by bacterial DNA may essentially contribute to the control of intestinal homeostasis.

There, however, seems to be no sufficient explanation for the strong protective qualities of TLR9 activation in colitis models as not only orally administered CpG-ODN but also systemic CpG-ODN application protects from intestinal inflammation. Another important epithelial cell-dependent mechanism might be the CpG/TLR9-induced secretion of bactericidal proteins [26] and/or induction of prostaglandin E_2_ [19, 27] both of which are considered as protective for the intestinal mucosa. In this case, the activation of epithelial cells may even occur from the basal membrane after systemic administration of CpG motifs.

Apart from these possible epithelial cell-mediated mechanisms we could identify a CpG/TLR9-mediated modulation of T-cell function in the CD4^+^ T-cell-dependent SCID transfer model of colitis in which colitis is induced in SCID recipients by adoptively transferred CD4^+^CD62L^+^ cells. Pretreatment of donor mice with CpG-ODN completely abolished colitis development in SCID recipients. Even more important, CD4^+^CD62L^+^ cells from donors which were exposed to CpG motifs had the capacity to inhibit the development of colitis when cotransferred with colitis-inducing CD4^+^CD62L^+^ cells from untreated donors, indicating a regulatory potential of CD4^+^CD62L^+^ cells from CpG-ODN treated donors [28]. In fact CD4^+^ T-cells from germ-free CpG-ODN-treated donor mice displayed increased PD-1 and FoxP3 expression as potential markers of regulatory T-cells [29].

Vice versa, transfer of cells from TLR9-deficient mice resulted in a much more severe intestinal inflammation compared to cells from wildtype controls [28]. This does not only confirm that CpG motifs can attenuate proinflammatory lymphocyte function but also underlines that the physiological TLR9/CpG interactions are necessary for immune homeostatic mechanisms such as controlling the proinflammatory potential among CD4^+^ T cells.

Concerning the further characterization of CD4^+^-dependent prophylactic effects of bacterial DNA we asked three main questions.

1. Is there a direct TLR9/CpG-interaction on CD4^+^ T-cells or is the effect mediated indirectly via dendritic cells or Bcells known to be constitutively TLR9-positive?
2. In the case of indirect effects; which cellular and/or molecular mediators play a role in the modulation of T-cell functions?
3. Is TLR9 activation alone sufficient to induce prophylactic effects or is it dependent on the coexistence of other bacterial products (as they are endogenously found in all colonized mice)?

Our recent results indicated that dendritic cells (DC) are essential for the mediation of prophylactic TLR9-dependent effects on T-cells. When DC-depleted donor mice were treated with CpG-ODN, the colitis-attenuating properties of transferred T-cells were absent in SCID hosts. In addition, CpG-ODN preincubation of purified CD4^+^ T-cells with dendritic cells but not with B cells or alone resulted in an effective reduction of their colitogenic potential after transfer in SCID hosts [30]. Important molecular mediators conferring protective CpG effects of bacterial DNA to T cells seem to be type-I interferons. When type-I interferons were neutralized in CpG-ODN-treated donor mice, transferred T-cells again induced severe colitis in SCID hosts. Vice versa IFN-*β* treatment of donor mice strongly reduced the colitogenic potential of transferred CD4^+^ T-cells. In addition to these T-cell dependent protective effects of CpG-ODN induced type-I interferons, IFN-*β* has been shown to mediate colitis-protective effects in a model in which inflammation is only dependent on innate immune responses [31].

The detailed mechanisms by which T-cells modulated by CpG motifs prevent colitis are still unclear. According to recent results it seems likely that increased secretion of IL-10 participates in protection from colitis after transfer of CD4^+^ T cells from CpG-ODN-treated donors [29].

## 4. Bacterial DNA in Colitis: Good, Bad, or Both?

Despite increasing insight in intestinal immune-modulating properties of TLR9 activation, it is still unclear which mechanisms explain the dichotomic effect in intestinal inflammation, including both protective and proinflammatory qualities. According to our results we favour the hypothesis that the preactivation status of the intestinal immune system essentially contributes to the outcome of TLR9 activation, being protective under healthy physiologic conditions but contributing to a further exacerbation in already established chronic colonic inflammation. A recent publication underlines that the outcome of TLR9-activation fundamentally differs depending on the anatomical location in the gastrointestinal tract. Hall et al. described a proinflammatory role of DC activated by TLR9 engagement even under healthy conditions. Such activated DC inhibited regulatory T-cells and activated Th_1_ and Th_17_ T-cells [32]. These proinflammatory effects, however, were restricted to the small intestine in which the number of resident bacteria strongly differs from that in the colon altering the physiologic microbial/immune system interaction and, thus, the status of immune activation.

It is important to note that these paradoxical effects are not restricted to TLR9-activation but have also been described for other TLR ligands and the bacterial flora in general.

It is well documented that commensals have the potential to trigger and perpetuate intestinal inflammation in various spontaneous colitis models like IL-10 deficient mice [33] which do not develop colitis when kept under germ-free conditions. This seems to be in contrast to recent results which indicate that the physiologic colonization of the gut with commensals is a necessary prerequisite for a properly working (mucosal) immune system [34–36]. In fact, the permanent interaction of commensals and their constituents with the mucosal immune system has been shown to exert strong protective functions concerning intestinal inflammation. Genetically engineered mice which are unable to respond to TLR4 or TLR2 ligands such as LPS or lipoteichoic acid developed a strikingly more severe intestinal inflammation after chemically induced colitis [37]. This has been explained by a reduced barrier function due to a lack of bacteria-induced mucosal secretion of defensins and mucins by paneth cells and intestinal epithelial cells. Beyond this impaired production of protective mucosal molecules, we could demonstrate—in line with the results in TLR9-deficient mice described above—that commensals/immune system interactions also contributed to a less aggressive behaviour of transferred naïve CD4^+^ T-cells in the SCID transfer model of colitis. Transfer of naïve CD4^+^ T-cells from germ-free donor mice to SPF-housed SCID recipients resulted in a much more severe intestinal inflammation than transfer of such cells from conventionally housed donors with a normal bacterial flora [29, 38].

**Figure 1:**
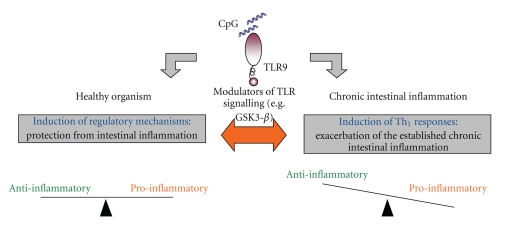
Whereas in a healthy organism under steady-state conditions CpG DNA/TLR9 activation favours the induction of regulatory mechanisms in chronic intestinal inflammation CpG/TLR9 interaction contributes to the perpetuation of chronic intestinal inflammation. The identification of possible regulators of TLR9 activation (e.g. GSK3-*β*) which have the potential to decide whether TLR activation results in a more tolerizing or proinflammatory response may help to explain these dichotomic effects. These regulators are interesting targets for therapeutic approaches.

The key helping to resolve these paradoxical effects may be a differential regulation of TLR signalling as shown in Figure 1. Recently, several molecules were identified which decide—depending on their state of activation—whether TLR-activation results in a strong proinflammatory response or a more tolerizing response [39]. For example, activated glycogen synthase kinase 3-*β* (GSK3-*β*) led to a strong TLR-dependent TNF-, IL-6-, and IL-12-production in DC whereas TLR-activation in DC with low levels of activated GSK3-*β* resulted in a predominant IL-10 production and production of only low levels of proinflammatory cytokines [40]. We could demonstrate that inhibition of GSK3-*β* ameliorated chronic intestinal inflammation in chronic DSS-induced colitis and even abrogated the proinflammatory effects of an additional CpG-ODN exposition seen in established chronic colitis [41]. Interestingly, human lamina propria cells displayed an increased production of proinflammatory cytokines upon TLR9-activation compared to non-IBD patients which could be abolished by blockade of GSK3-*β* [41]. This underlines a possible relevance for this TLR-regulating molecule also in the human situation.

Targeting key modulators of TLR signalling such as GSK3-*β* may represent a promising option for future therapeutic approaches. While current therapeutic principles just more or less effectively inhibit proinflammatory immune responses at the cost of an increased risk for serious infections, the restoration of the physiologic equilibrium between effectors and inhibitors may help to reestablish the friendly coexistence of luminal bacteria and the intestinal immune system. This should not only result in reduced side effects but may even have the potential to achieve long-lasting remission.
